# War Gratuities for Regular Officers

**Published:** 1919-04-05

**Authors:** 


### WAR GRATUITIES FOR REGULAR OFFICERS.

At least twice since the date of the Armistice it has been officially announced in the columns of the newspapers that the question of a gratuity to regular officers of the Army for their services in the war is "under consideration." "Who gains quickly gains twice over." This old saying might well be realised by those authorised to make the grant. The regular officer bore the heat and burden, or the cold and burden, of the first months of the war. The regular officer made the new armies. The regular officer died by the thousand to stem the German's first fierce rush. The regular officer has not agitated, nor has any one else agitated for him. But he has felt the war prices as much as any other and more, for he had made a very bad bargain with the British people when he accepted the pay the British nation offered him. He has had to maintain his wife and family, if he were married in England, without any consideration or separation allowance. He has had to replace his uniform and equipment at war prices, but he has had neither rise nor bonus. Even when the torpedoes of the Hun or the exigencies of retreat have deprived him of the kit he carried with him to the war, only the most meagre minimum has been allowed him to replace it, a minimum that has invariably left him heavily out of pocket. And now he is sardonically suffering, with many a bitter jest, that the consideration of his gratuity is only consideration of how small it can possibly be made. He will not agitate. He will not strike. But he will not feel he has been well treated if the measure meted out to him is very scanty compared to that given to men who have done no more than he in the war area, and to the others who have risked much less than he in the works on this side of the English Channel. Now is it likely that he will strongly urge his sons to follow his footsteps in a service of such poor rewards? The fact is that the whole position of the officers of the Regular Army requires overhauling. They have not been treated justly nor generously in the past. Nothing would better become the nation now than a generous pecuniary acknowledgment of the magnificent services the regular officers have rendered it—ungrudgingly rendered it—during this war. This should be done promptly. The sooner the better. Thereafter the nation should decide on what is a proper pay for men of good education, ability, tact, courage, and power of leadership such as it requires and will require to lead the men that will guard its shores, uphold its honour, and enforce its rights in all quarters of the earth. This payment should be generous, and not a sum which many a small shopkeeper would consider a poor return for his huckstering. Demands may be just or unjust. If the demand would be just if made it is better to be generous and make the offer before the demand comes. The British nation always puts all blame for anything not creditable to itself on its Government. That will not do. The nation has the Government it deserves and is responsible for the acts of its agent as much as any private firm. The nation must not allow it to be thought that it will not give justice even till it has a pistol at its head and that then it will give anything asked. That now seems to be the opinion of large sections of the community. The regular officer is little likely to present the pistol. That is the stronger reason for granting him justice.

